# One-tissue compartment model for myocardial perfusion quantification with N-13 ammonia PET provides matching results: A cross-comparison between Carimas, FlowQuant, and PMOD

**DOI:** 10.1007/s12350-021-02741-4

**Published:** 2021-08-18

**Authors:** Sergey V. Nesterov, Roberto Sciagrà, Luis Eduardo Juarez Orozco, John O. Prior, Leonardo Settimo, Chunlei Han, Emmanuel Deshayes, Robert A. deKemp, Darja V. Ryzhkova, Kilem L. Gwet, Juhani M. Knuuti

**Affiliations:** 1 Turku PET Centre, University of Turku and Turku University Hospital, Turku, Finland; 2 University of Florence, Florence, Italy; 3 University of Lausanne, Lausanne, Switzerland; 4 National Cardiac PET Center, University of Ottawa Heart Institute, Ottawa, Canada; 5 Almazov Federal Heart, Blood and Endocrinology Centre, St. Petersburg, Russia; 6 Advanced Analytics LLC, Gaithersburg, MD USA; 7 IM Sechenov Institute of Evolutionary Physiology and Biochemistry RAS, St. Petersburg, Russia; 8 Regional Cancer Institute of Montpellier (ICM) - Val d’Aurelle, Montpellier, France

**Keywords:** Myocardial perfusion imaging, positron emission tomography, N-13 ammonia, quantitative imaging, absolute quantification, Imaging software, agreement, reproducibility, standardization of PET

## Abstract

**Purpose:**

To cross-compare three software packages (SPs)—Carimas, FlowQuant, and PMOD—to quantify myocardial perfusion at global, regional, and segmental levels.

**Materials and Methods:**

Stress N-13 ammonia PET scans of 48 patients with HCM were analyzed in three centers using Carimas, FlowQuant, and PMOD. Values agreed if they had an ICC > 0.75 and a difference < 20% of the median across all observers.

**Results:**

When using 1TCM on the global level, the agreement was good, and the maximum difference between 1TCM MBF values was 17.2% (ICC = 0.83). On the regional level, the agreement was acceptable except in the LCx region (25.5% difference, ICC = 0.74) between FlowQuant and PMOD. Carimas-1TCM agreed well with PMOD-1TCM and FlowQuant-1TCM. Values obtained with FlowQuant-1TCM had a somewhat lesser agreement with PMOD-1TCM, especially at the segmental level.

**Conclusions:**

The global and regional MBF values (with one exception) agree well between the different software packages. There is significant variability in segmental values, mainly located in the LCx region and segments. Out of the studied tools, Carimas can be used interchangeably with both PMOD and FlowQuant for 1TCM implementation on all levels—global, regional, and segmental.

**Supplementary Information:**

The online version contains supplementary material available at 10.1007/s12350-021-02741-4.

## Introduction

Absolute quantification of myocardial blood flow (MBF) in mL·min^−1^·g^−1^ of myocardial tissue with dynamic PET imaging constitutes an essential tool for clinicians. It can provide relevant information complementary to relative myocardial perfusion imaging.^1^,^2^ Currently, optimized acquisition protocols, incremental computational power, and fast image reconstruction enable list-mode-acquired PET imaging and myocardial perfusion quantification (MPQ) to be applied in clinical routine.^3^

There has been a continuous effort to harmonize imaging workflow. One factor that remains unconstrained is the software package (SP) variability.^1^,^4^,^5^ In 2014, we published a comparative study^4^ that considered ten SPs for Rb-82 PET. Results showed that MBF and MFR values obtained with different SPs could differ by a factor of two or more. However, the agreement was satisfactory when only one specific model—a one-tissue compartment model (1TCM,^6^)—was applied.

Nitrogen-13 ammonia represents another widely used perfusion PET tracer. It has a short half-life and therefore requires an on-site cyclotron for its clinical implementation. It has also demonstrated more favorable extraction and retention kinetics and quantification precision than Rb-82.^7^ Previous data on SPs comparison for N-13 ammonia MPQ were reported^8^ in patients with known or suspected coronary artery disease (CAD).

Furthermore, MPQ has been performed in pathological conditions beyond CAD, such as cardiomyopathies. Studies in hypertrophic cardiomyopathy (HCM) demonstrated hampering of the vasodilator reserve and microvascular dysfunction, impacting the prognosis for the patients.^9^,^10^ Despite these efforts at characterizing perfusion abnormalities in HCM, there is a lack of reports addressing the MPQ agreement considering this particular pathology.

Hence, the present study aimed to cross-compare stress MPQ with N-13 ammonia between three SPs—Carimas, FlowQuant, and PMOD—which provide different implementations of the same one-tissue compartment model (1TCM). The MPQ agreement evaluation was performed in the patients with HCM at the three levels of segmentation—global, regional, and segmental (based on the 17-segment AHA model.^11^)

## Materials and Methods

The study group consisted of 48 patients with known HCM referred to PET MPQ (patient characteristics are in Table 1).

**Table 1:** Population characteristics

*n*	48
No. of males (% of total)	30 (63%)
Age, years. (range)	44 ± 15.4 (16–79)
Weight, kg (range)	70 ± 11.6 (4–98)
Body mass index, kg/m^2^ (range)	24.1 ± 3.43 (18.3–32.9)
Symptoms	
Angina	4 (8%)
Dyspnea	20 (42%)
Syncope	5 (10%)

Values are *n* (%), or arithmetic means ± SD

### Image Acquisition

All PET studies were performed at the Nuclear Medicine Unit of the Department of Clinical Physiopathology of the University of Florence (Italy) according to the corresponding routine clinical practice. The local ethics committee approved the study protocol, and written informed consent was obtained from each patient before the study. Only stress studies were performed to lessen the radioactivity burden. Patients were positioned in the PET scanner (GE Advance), and a transmission scan was recorded. Then, dipyridamole was administered intravenously (0.56 mg·kg^−1^ of body weight) over 4 minutes. After 3 minutes of dipyridamole infusion, a bolus of 370 MBq of nitrogen-13 ammonia (N-13 ammonia) diluted in 10 mL saline solution was injected intravenously over 15 to 20 seconds. A dynamic scan (2D mode) was acquired for 4 minutes, followed by a prolonged static acquisition of 15 minutes. Dynamic images were reconstructed into 15 frames (12 × 10 seconds, 2 × 30 seconds, and 1 × 60  seconds).

### Image Analysis

The reconstructed stress images were delivered to three centers in Finland, Italy, and Switzerland. In each center, an investigator used one software package—Carimas in Finland, PMOD in Italy, FlowQuant in Switzerland—and, by the rules of this project, had been blinded to results of the image analysis of the other investigators before they shared their findings.

The image analysis process in all packages consisted of image reorientation, LV myocardium and cavity segmentation, and tracer kinetic modeling (TKM). All three SPs implemented 1TCM described by DeGrado et al.^12^

Carimas has semiautomatic segmentation at work but requires the operator interaction for TKM. FlowQuant enables automatic reorientation and segmentation with optional user adjustment. PMOD performs automatic reorientation and segmentation. For details of workflows in each of the SPs, see(4, Appendix).

The image analysis resulted in estimated values for stress MBF on global, regional, and segmental levels. At the global level, the package provided the average LV value and, at the regional level, values for the three vascular territories—LAD, LCx, and RCA—in the regions of coronary arteries using a default template. The segmental level corresponded to the 17-segment AHA standard model.^11^

### Statistical Analysis

In the study, we compared the estimates from the 1TCM across all its three implementations.

As the overall number of compared sets exceeded two, we could not use the standard approach to measure agreement proposed by Bland and Altman,^13^ and we applied a linear mixed model for the repeated measures (MMRM)^14^ to the dataset. The statistical model output included two main agreement metrics—intraclass correlation coefficient (ICC) and the difference between the values from the implemented TCMs—both calculated pairwise.

The pairwise agreement between compared stress MBF values was considered acceptable if the difference between them was less than 20% of the corresponding median across all investigators and if the corresponding ICC was equal or over 0.75. The criterion for ICCs we based on the literature data.^15^ The standard for the differences we introduced in earlier work^4^—we considered the cutoff value of 20% acceptable based on previous work in the field^16^ and the reported variability of within-subject perfusion estimates.^17^ We expressed the difference between stress MBF values as a percent of corresponding medians to unify the scale through all the measured parameters.

When required, the significance of differences between paired groups was calculated with a two-tailed *t*-test, assuming unequal variance.

### Biplot Analysis

To visualize the results of the cross-comparisons, we used a custom biplot,^4^ relating two criteria: the difference and the ICC values of the compared pairs. In this biplot, the *X*-axis shows pairwise differences between the model values, and the *Y*-axis shows corresponding pairwise values of 1 minus ICC. The origin (*x* = 0 and *y* = 0) is the point of identity between the compared values with no difference, and ICC is equal to 1. Thus, values further from the origin agree less: either showing the increased difference, the reduced ICC, or both. These predefined criteria of the agreement are plotted as a rectangular region on the biplot covering all the acceptable cross-comparisons.

## Results

### Absolute Values of Stress MBF

All MBF estimates, as well as calculated median values, are in Table 2. In general, FlowQuant had the lowest and PMOD—the highest MBF values obtained with the 1TCM.

**Table 2:** Stress MBF values

	Carimas-1TCM	FlowQuant-1TCM	PMOD-1TCM	Max/Min ratio	Median
Global	1.47 ± 0.52	**1.46 ± 0.42**	**1.73 ± 0.56**	1.18	1.55
LAD	1.32 ± 0.49	**1.31 ± 0.39**	**1.50 ± 0.52**	1.15	1.38
LCx	1.81 ± 0.63	**1.64 ± 0.46**	**2.09 ± 0.71**	1.27	1.77
RCA	1.57 ± 0.59	**1.51 ± 0.51**	**1.73 ± 0.61**	1.15	1.59
Seg1 *(basal anterior)*	1.64 ± 0.58	**1.53 ± 0.47**	**1.79 ± 0.76**	1.17	1.66
Seg2 *(basal anteroseptal)*	1.38 ± 0.49	**1.24 ± 0.44**	**1.52 ± 0.56**	1.23	1.38
Seg3 *(basal inferoseptal)*	1.52 ± 0.59	**1.41 ± 0.53**	**1.62 ± 0.61**	1.15	1.47
Seg4 *(basal inferior)*	1.74 ± 0.69	**1.73 ± 0.57**	**2.00 ± 0.72**	1.16	1.81
Seg5 *(basal inferolateral)*	1.88 ± 0.70	**1.59 ± 0.49**	**2.17 ± 0.80**	1.36	1.78
Seg6 *(basal anterolateral)*	1.90 ± 0.68	**1.72 ± 0.53**	**2.20 ± 0.86**	1.28	1.82
Seg7 *(mid anterior)*	**1.72 ± 0.64**	**1.45 ± 0.46**	1.66 ± 0.61	1.19	1.58
Seg8 *(mid anteroseptal)*	1.43 ± 0.53	**1.24 ± 0.42**	**1.44 ± 0.53**	1.16	1.33
Seg9 *(mid inferoseptal)*	1.52 ± 0.58	**1.44 ± 0.52**	**1.68 ± 0.64**	1.17	1.54
Seg10 *(mid inferior)*	1.78 ± 0.74	**1.65 ± 0.58**	**1.82 ± 0.75**	1.10	1.68
Seg11 *(mid inferolateral)*	1.93 ± 0.73	**1.63 ± 0.48**	**2.12 ± 0.77**	1.30	1.79
Seg12 *(mid anterolateral)*	1.98 ± 0.70	**1.74 ± 0.50**	**2.10 ± 0.73**	1.21	1.86
Seg13 *(apical anterior)*	**1.44 ± 0.57**	**1.38 ± 0.43**	1.40 ± 0.53	1.04	1.38
Seg14 *(apical septal)*	**1.29 ± 0.50**	**1.17 ± 0.43**	1.28 ± 0.52	1.10	1.19
Seg15 *(apical inferior)*	1.43 ± 0.55	**1.30 ± 0.48**	**1.44 ± 0.62**	1.11	1.31
Seg16 *(apical lateral)*	1.59 ± 0.64	**1.50 ± 0.46**	**1.63 ± 0.66**	1.09	1.51
Seg17 *(apex)*	1.20 ± 0.53	**1.16 ± 0.38**	**1.23 ± 0.56**	1.06	1.12

Values are mean ± SD (n = 48); median values are calculated for n = 144 in 1TCM. Minimum (Min) and maximum (Max) values in each row for 1TCM are in bold

### Agreement Between the Global and Regional Values Using 1TCMs

**Figure 1:**
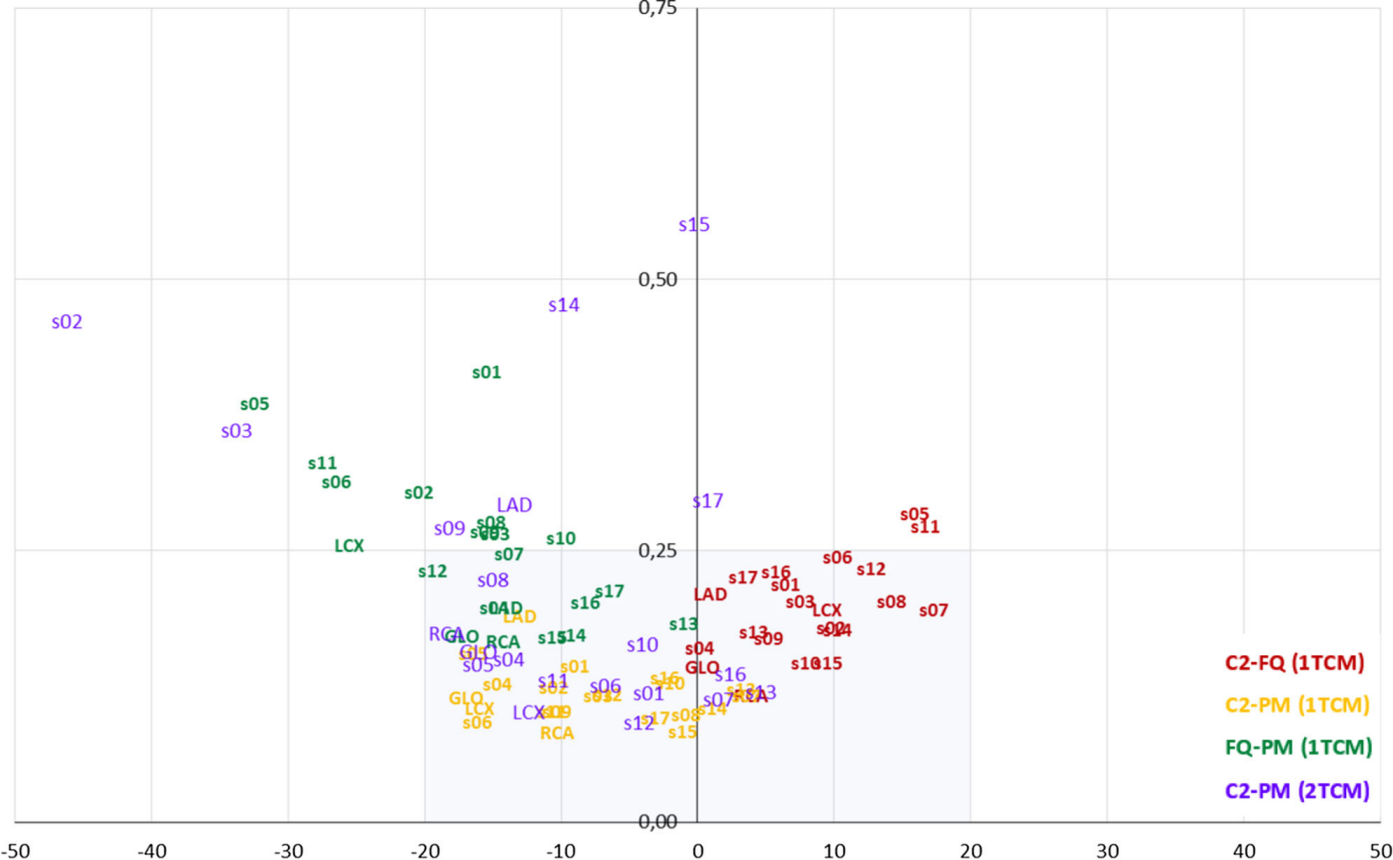
Comparison of stress MBF values on global and regional levels. The *X*-axis is the difference in MBF values expressed in percent of corresponding medians; *Y*-axis is 1-ICC. The shaded iris area limits the acceptable agreement: ± 20% of the median value on the *X*-axis and ICCs over 0.75 on the *Y*-axis. The chart element corresponds to the comparison in the left ventricle area of the same name. *C2*, Carimas; *FQ*, FlowQuant; *PM*, PMOD

Figure 1 presents comparisons between MBF values obtained with the same 1TCMs in the studied SPs at the global and regional levels. The maximum difference between MBF values at the global level was in the acceptable range—17.2% (ICC = 0.83) between FlowQuant and PMOD. Regionally, the values obtained with different SPs agreed well except the LCX region between FlowQuant and PMOD that had a maximum difference of 25.5% of the median value (acceptable difference < 20%) at ICC = 0.74 (acceptable > 0.75).

Values obtained with Carimas were almost identical to FlowQuant on a global level—the difference—0.3% and ICC = 0.86. On a regional level, the maximum difference between these two SPs was 9.5% in LCx (ICC = 0.80), and the worst ICC was 0.79 in LAD (the corresponding difference—1%).

### Agreement Between the Segmental Values Using 1TCMs

**Figure 2:**
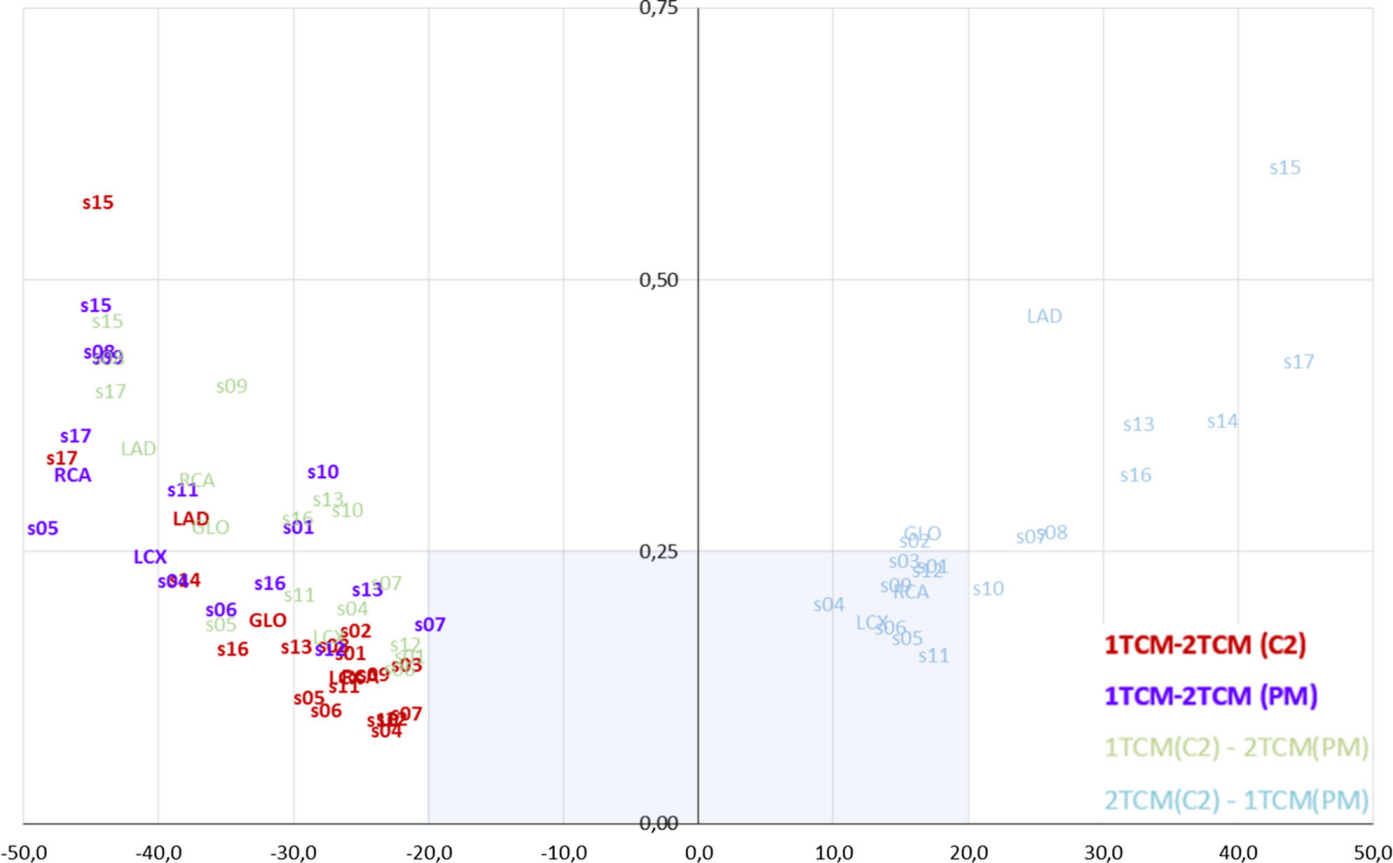
Comparison of stress MBF values on the segmental level. The *X*-axis is the difference in MBF values expressed in percent of corresponding medians; *Y*-axis is 1-ICC. The shaded iris area limits the acceptable agreement: ± 20% of the median value on the *X*-axis and ICCs over 0.75 on the *Y*-axis. The chart element corresponds to the comparison in the same name’s left ventricle area (e.g., ‘15’ stands for comparison in segment 15). *C2*, Carimas; *FQ*, FlowQuant; *PM*, PMOD

The agreement at the segmental level is in Figure 2. The maximum difference between Carimas and FlowQuant was 17.4% in segment 7 (mid anterior) (ICC = 0.80). Yet, there were two comparisons with ICCs less than 0.75:0.72 in segment 5 (basal inferolateral) (corresponding difference—16.0%) and 0.73 in segment 11 (mid inferolateral) (corresponding difference—16.7%).

Matching the trend of the regional values, the segmental values obtained with FlowQuant had a lesser agreement with PMOD. Nine out of 17 segmental comparisons did not fulfill one or both the agreement criteria. On the segmental level, the maximum difference was in segment no. 5 (basal inferolateral)—32.4% (ICC = 0.61); minimum—in segment no. 13 (apical anterior)—0.9% (ICC = 0.82).

**Figure 3:**
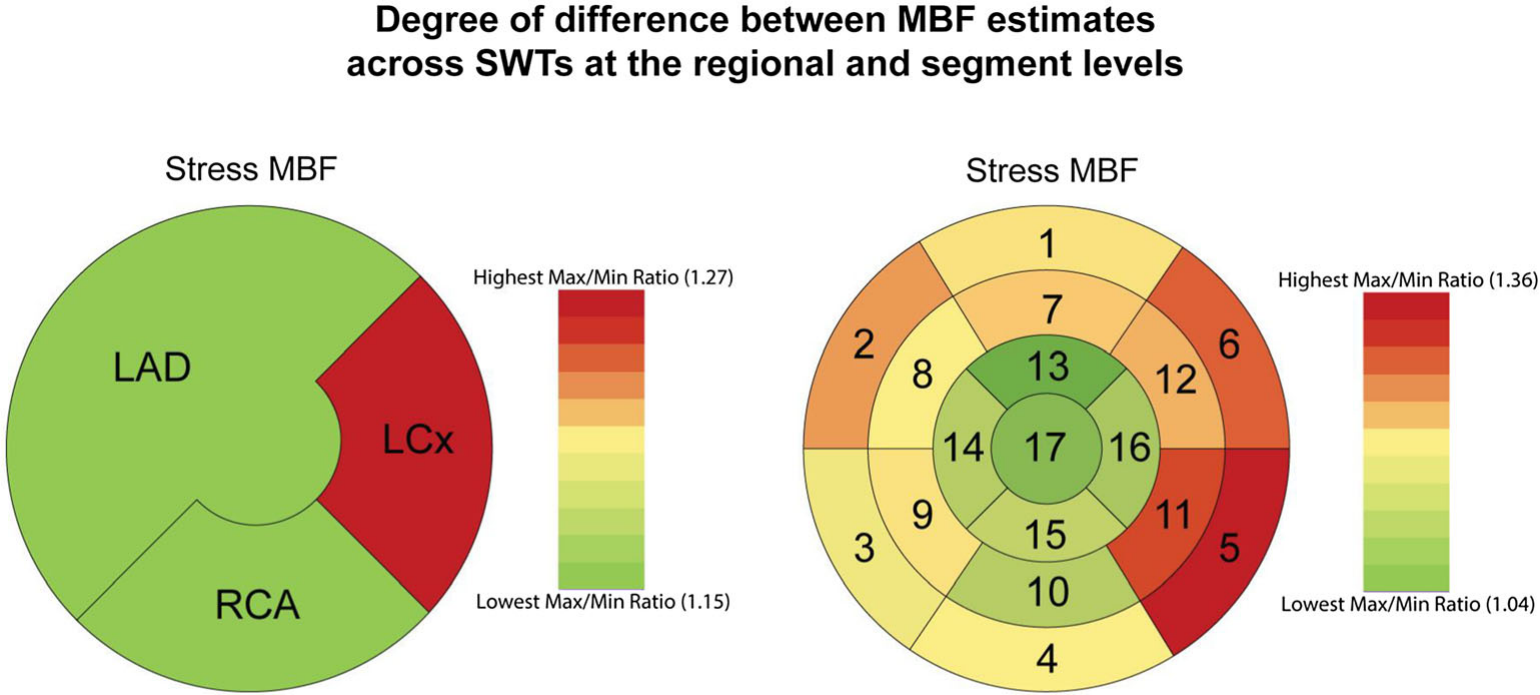
Polar maps demonstrating the differences in estimated MBF values across SPs depicted across the levels of segmentation of the myocardium.

The differences in estimated MBF values across SPs and the anatomical location of each segment are in Figure 3

## Discussion

The present study compared three SPs for myocardial perfusion quantification with N-13 ammonia—Carimas, FlowQuant, and PMOD—in a population of 48 patients with HCM. The comparison was made on 48 pharmacological stress images, and the obtained MBF estimates were compared on three sequential levels—global, regional, and segmental. All three tools were analyzed for their implementation of the 1TCM described in.^12^

Of the MBF values obtained, PMOD generally—global, all regional, and 14 of 17 segmental—provided the highest values, while FlowQuant provided the lowest. In Carimas, flow values were generally between FlowQuant and PMOD, which explained why Carimas had an excellent agreement with both other tools. The observed differences unlikely come from the implementation of the models but rather from image processing by the software—reorientation and segmentation, which means that our efforts to standardize the approach must be directed to these areas as well.

In global MBF using 1TCM, all three SPs had a good agreement. Also, at the regional level, the agreement was acceptable except for the LCx region—there was a 25.5% difference between PMOD and FlowQuant. Not surprisingly, the differences were pronounced at the segmental level, especially between PMOD and FlowQuant. In contrast, the agreement was excellent when comparing Carimas with PMOD and FlowQuant on all the three studied levels down to the segmental. Although the comparison between PMOD and FlowQuant showed overall good agreement, we cannot recommend using them interchangeably on the segmental level.

We found more disagreement in the regional and segmental LCx estimates, which is interesting since N-13 ammonia has recurrently documented variable lateral wall perfusion defects. These “artifacts” are common to a degree in which they have been considered a “normal variant.” However, the fundamental nature of this phenomenon is still unknown. We hypothesize that this may arise due to spatial detection issues, extracardiac uptake, and ventricle location. To which degree the described variability between SPs plays a role is yet to be elucidated.

Our approach to the different quantification levels from global to segmental level was a distinctive feature of this study. Generally, researchers cross comparing myocardial PET software avoid the segmental level, the exclusions being our studies of O-15 water^18^ and C-11 acetate.^19^ Yet, to envision the “clinical reality” of MPQ, we cannot bypass this level. It provides us with more information on the myocardium, which may be important when important are regional data, as it is with CAD

Ideally, SPs should convey (nearly) identical results in MPQ, which would be expected considering that any quantification results, or variation therein, are bound to be attributed to several (patho)physiological processes and interpreted accordingly in the diagnostic process. Nevertheless, in reality, each SP implements particular reorientation, segmentation, and sampling methods for the LV myocardium and blood-pool activity to obtain input curves. Discrepancies in absolute parameter quantification are likely to undermine efforts that call for pooling results, comparison of estimates across populations, and development of sensitive cutoff values, as we earlier reported the possible problems.^20^,^21^

Progress in the field of cardiac PET is possible when clinical protocols are aligned with interpretation protocols. The concept of having a locally tested PET system that takes care of all the steps from the scanner to the dedicated analysis (5,20, and Lance K. Gould, personal communication) indeed enables pooling but only locally so. We deem that global standardization is a goal for cardiac PET. This study focused on the analysis and interpretation by comparing some SPs’ performance in patients with HCM, hopefully, to enable better pooling of results obtained from studies using each of these tools independently. This pooling is fundamental to take cardiac PET imaging to the next step of adopting and demonstrating efficiency.

There are obvious limitations in this study. This analysis focused only on three SPs and cannot inform about the general discrepancies between the various SPs. No reference standard was available in this study, so we do not know the true MBF in our patients and cannot tell which SP was the most accurate. The study population had HCM. Although likely, we do not know if the results can be generalized to patients with CAD or suspected of CAD. The images were acquired on a particular 2D PET scanner. The results could be different with a modern system with better image statistics.

## New Knowledge Gained

The global and regional MBF values (N-13 ammonia PET MPQ) agree well between the different software packages implementing 1TCM; segmental values show significant variability. Carimas can be used interchangeably with both PMOD and FlowQuant on all levels.

## Conclusions

1. The global and regional MBF values (with one exception) agree well between the different software packages,
2. However, there is significant variability in segmental values, mainly located in the LCx region and segments.
3. Out of the studied tools, Carimas can be used interchangeably with both PMOD and FlowQuant for 1TCM implementation on all levels—global, regional, and segmental.

## Supplementary Information

Below is the link to the electronic supplementary material.

Supplementary file1 (PPTX 3187 KB)

Supplementary file2 (M4A 6366 KB)

## Abbreviations

1TCM: One-tissue compartment model
HCM: Hypertrophic cardiomyopathy
ICC: Intraclass correlation coefficient
LV: Left ventricle
MBF: Myocardial blood flow
MFR: Myocardial flow reserve
MMRM: Linear mixed model for repeated measures
MPQ: Myocardial perfusion quantification
SP: Software package
TKM: Tracer kinetic modeling

## References

[1] Saraste A, Kajander S, Han C, Nesterov SV, Knuuti J (2012). PET: Is myocardial flow quantification a clinical reality?. J Nucl Cardiol.

[2] Bengel FM, Higuchi T, Javadi MS, Lautamäki R (2009). Cardiac positron emission tomography. J Am Coll Cardiol.

[3] Kajander S, Joutsiniemi E, Saraste M, Pietila M, Ukkonen H, Saraste A, Sipila HT, Teräs M, Mäki M, Airaksinen M, Hartiala J, Knuuti J (2010). Cardiac positron emission tomography/computed tomography imaging accurately detects anatomically and functionally significant coronary artery disease. Circulation.

[4] Nesterov SV, Deshayes E, Sciagrà R, Settimo L, Declerck JM, Pan X-B (2014). Quantification of myocardial blood flow in absolute terms using 82Rb PET imaging. JACC Cardiovasc Imaging.

[5] Nesterov SV, Lee BC, Moody JB, Slomka P, Han C, Knuuti JM (2016). The status and future of PET myocardial blood flow quantification software. Ann Nucl Cardiol.

[6] Lortie M, Beanlands RSB, Yoshinaga K, Klein R, Dasilva JN, deKemp R (2007). Quantification of myocardial blood flow with 82Rb dynamic PET imaging. Eur J Nucl Med Mol Imaging.

[7] Renaud JM, Mylonas I, McArdle B (2014). Clinical interpretation standards and quality assurance for the multicenter PET/CT trial: 82Rb as an alternative radiopharmaceutical for myocardial imaging. J Nucl Med.

[8] Slomka PJ, Alexanderson E, Jácome R, Jiménez M, Romero E, Meave A, Le Meunier L, Dalhbom M, Berman DS, Germano G, Schelbert H (2012). Comparison of clinical tools for measurements of regional stress and rest myocardial blood flow assessed with 13N-ammonia PET/CT. J Nucl Med.

[9] Camici PG, Chiriatti G, Lorenzoni R (1991). Coronary vasodilation is impaired both in hypertrophied and non-hypertrophied myocardium of patients with hypertrophic cardiomyopathy: a study with 13N-ammonia and positron emission tomography. J Am Coll Cardiol.

[10] Cecchi F, Olivotto I, Gistri R, Lorenzoni R, Chiriatti G, Camici PG (2003). Coronary microvascular dysfunction and prognosis in hypertrophic cardiomyopathy. N Engl J Med.

[11] Cerqueira MD, Weissman NJ, Dilsizian V, Jacobs AK, Kaul S, Warren K (2002). Standardized myocardial segmentation and nomenclature for tomographic imaging of the heart. A statement for healthcare professionals from the Cardiac Imaging Committee of the Council on Clinical Cardiology of the American Heart Association. Circulation.

[12] DeGrado TR, Hanson MW, Turkington TG, Delong DM, Brezinski DA, Vallée JP (1996). Estimation of myocardial blood flow for longitudinal studies with 13N-labeled ammonia and positron emission tomography. J Nucl Cardiol.

[13] Bland JM, Altman DG (1986). Statistical methods for assessing agreement between two methods of clinical measurement. Lancet.

[14] Davis CS (2003). Statistical Methods for the Analysis of Repeated Measurements.

[15] Rosner B (2011). Fundamentals of biostatistics.

[16] Efseaff M, Klein R, Ziadi MC, Beanlands RS, deKemp R (2012). Short-term repeatability of resting myocardial blood flow measurements using rubidium-82 PET imaging. J Nucl Cardiol.

[17] Kitkungvan D, Johnson NP, Roby AE, Patel MB, Kirkeeide R, Gould KL (2017). Routine clinical quantitative rest stress myocardial perfusion for managing coronary artery disease: clinical relevance of test-retest variability. JACC Cardiovasc Imaging.

[18] Nesterov SV, Han C, Mäki M, Kajander S, Naum AG, Helenius H (2009). Myocardial perfusion quantitation with 15O-labelled water PET: high reproducibility of the new cardiac analysis software (Carimas). Eur J Nucl Med Mol Imaging.

[19] Nesterov SV, Turta O, Han C, Maki M, Lisinen I, Tuunanen H, Knuuti J (2015). C-11 acetate has excellent reproducibility for quantification of myocardial oxidative metabolism. Eur Heart J.

[20] Nesterov SV, Knuuti J (2018). How accurate is the accuracy?. J Nucl Cardiol.

[21] Nesterov SV, Knuuti J (2020). 82Rb-PET MPQ: Do normal values exist?. J Nucl Cardiol.

