# Supplementary material for: One-tissue compartment model for myocardial perfusion quantification with N-13 ammonia PET provides matching results: A cross-comparison between Carimas, FlowQuant, and PMOD
Source: J Nucl Cardiol. 2021 Aug 18;29(5):2543–50. doi: 10.1007/s12350-021-02741-4 (PMC9553813; doi:10.1007/s12350-021-02741-4)
Supplement: Supplementary file 1 — Supplementary file1 (PPTX 3187 KB) [file 12350_2021_2741_MOESM1_ESM.pptx]

## Slide 1
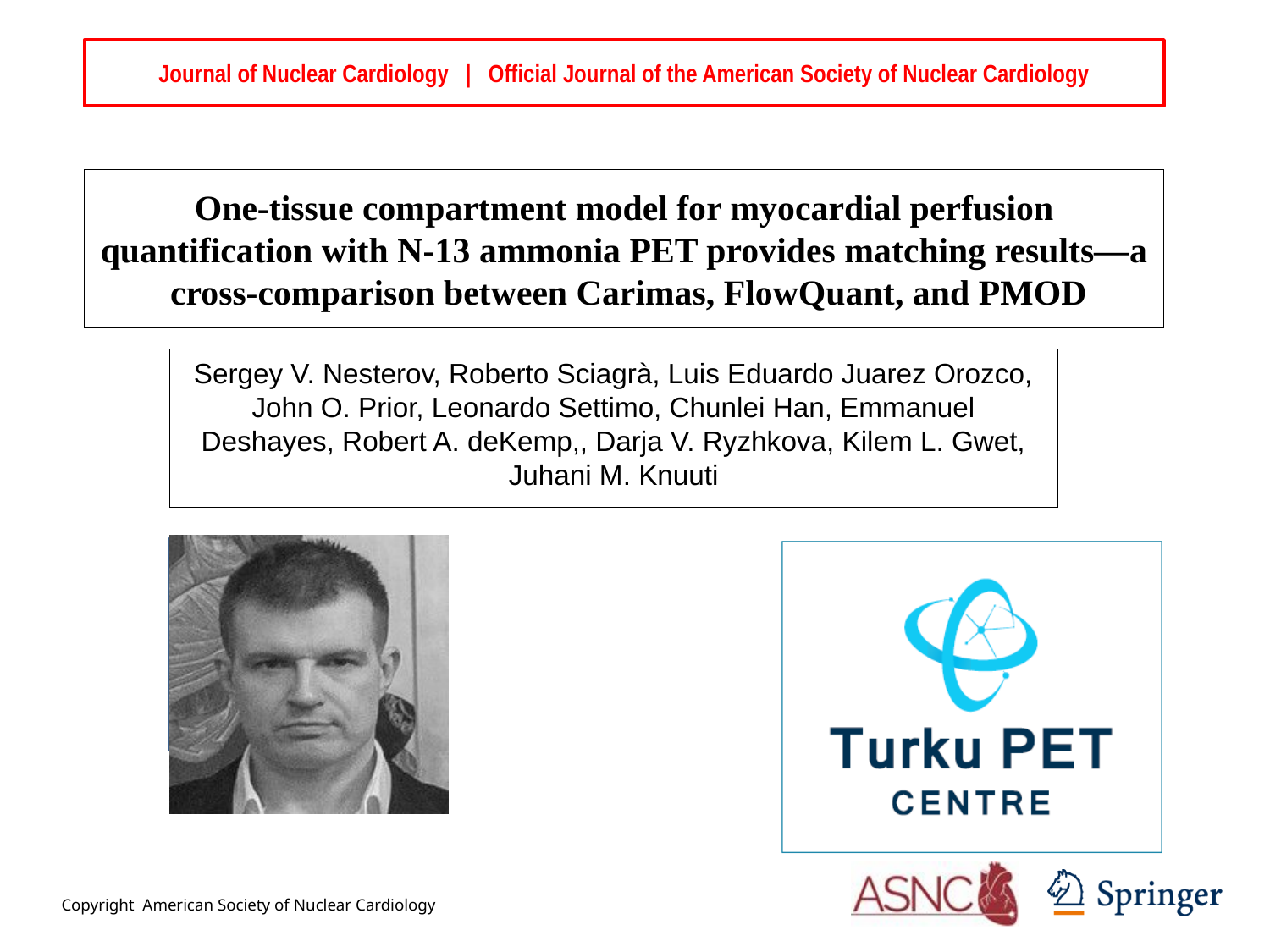

Journal of Nuclear Cardiology | Official Journal of the American Society of Nuclear Cardiology
# One-tissue compartment model for myocardial perfusion quantification with N-13 ammonia PET provides matching results—a cross-comparison between Carimas, FlowQuant, and PMOD
Sergey V. Nesterov, Roberto Sciagrà, Luis Eduardo Juarez Orozco, John O. Prior, Leonardo Settimo, Chunlei Han, Emmanuel Deshayes, Robert A. deKemp,, Darja V. Ryzhkova, Kilem L. Gwet, Juhani M. Knuuti
Head shot of author
required
Institution
Picture/Logo
Optional
Copyright American Society of Nuclear Cardiology

## Slide 2
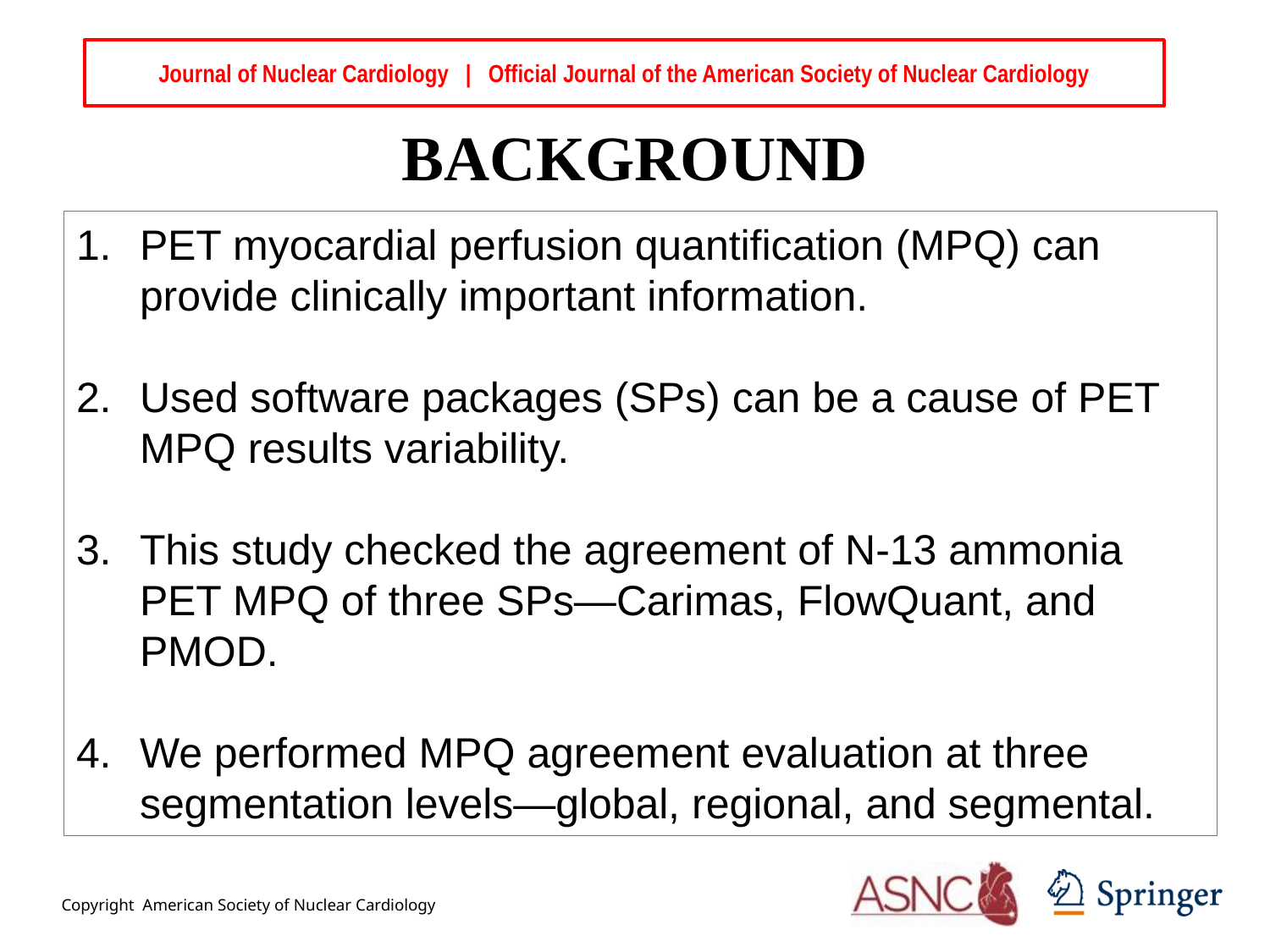

Journal of Nuclear Cardiology | Official Journal of the American Society of Nuclear Cardiology
# BACKGROUND
PET myocardial perfusion quantification (MPQ) can provide clinically important information.
Used software packages (SPs) can be a cause of PET MPQ results variability.
This study checked the agreement of N-13 ammonia PET MPQ of three SPs—Carimas, FlowQuant, and PMOD.
We performed MPQ agreement evaluation at three segmentation levels—global, regional, and segmental.
Copyright American Society of Nuclear Cardiology

## Slide 3
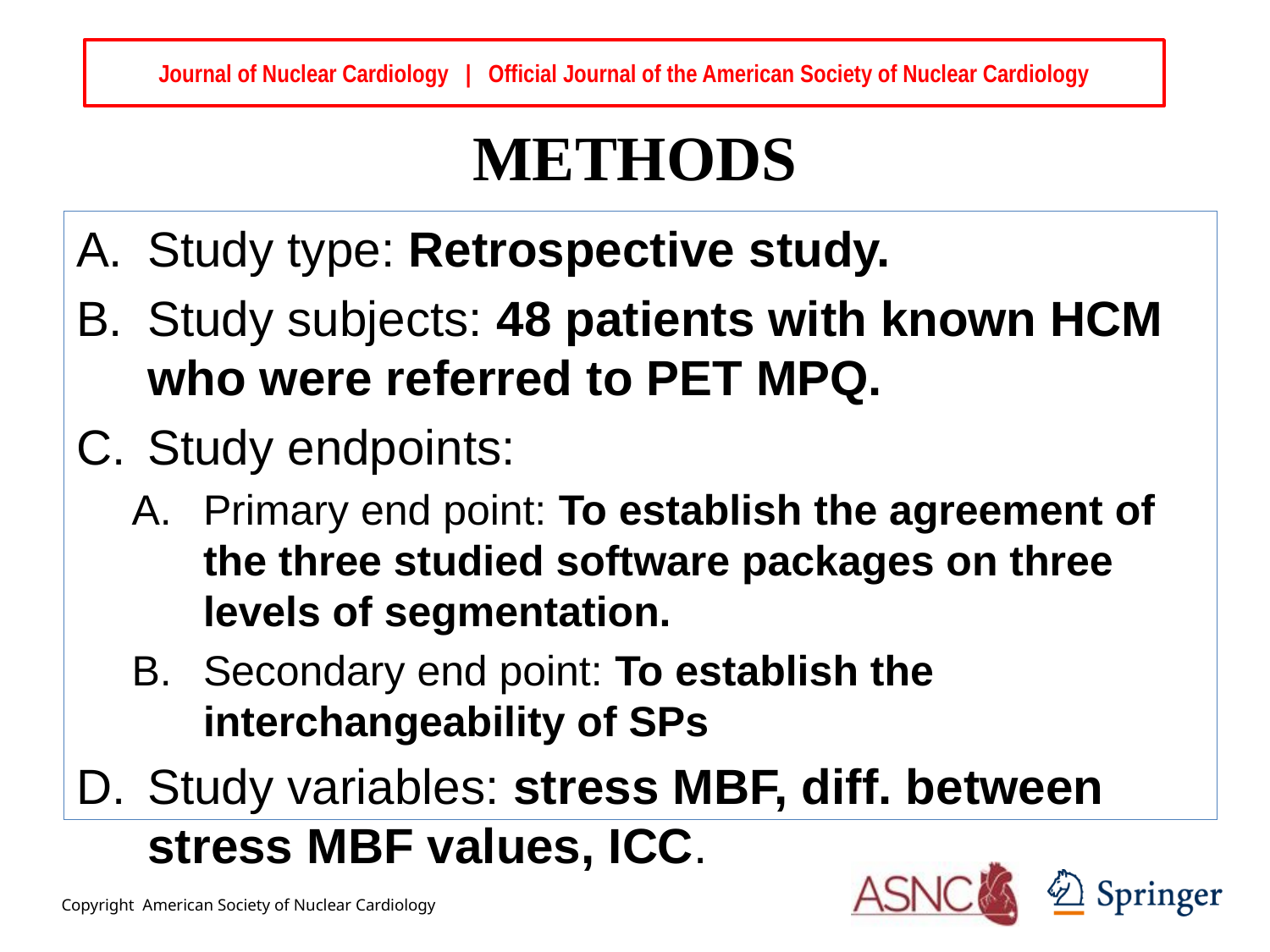

Journal of Nuclear Cardiology | Official Journal of the American Society of Nuclear Cardiology
# METHODS
Study type: Retrospective study.
Study subjects: 48 patients with known HCM who were referred to PET MPQ.
Study endpoints:
Primary end point: To establish the agreement of the three studied software packages on three levels of segmentation.
Secondary end point: To establish the interchangeability of SPs
Study variables: stress MBF, diff. between stress MBF values, ICC.
Copyright American Society of Nuclear Cardiology

## Slide 4
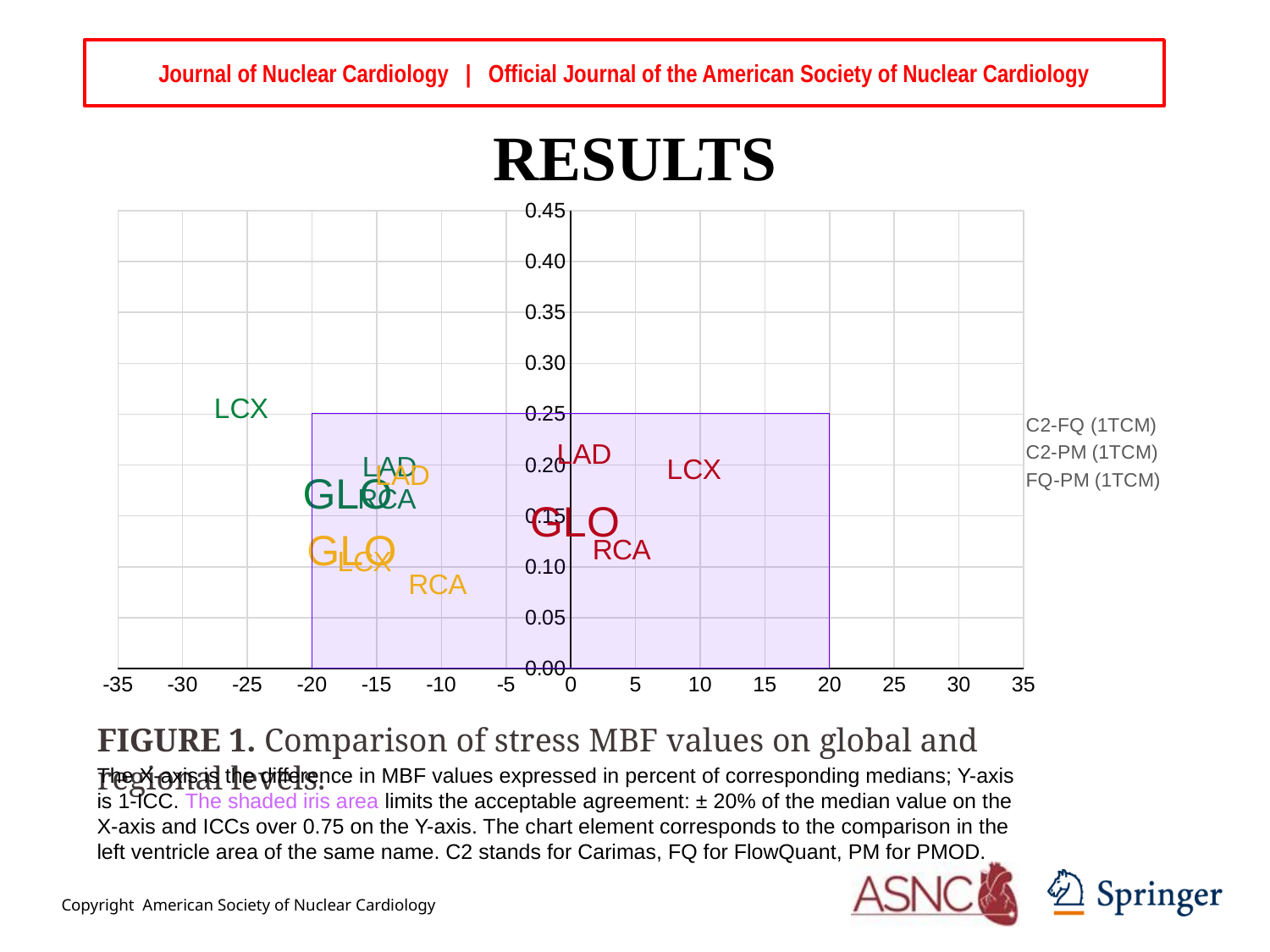

Journal of Nuclear Cardiology | Official Journal of the American Society of Nuclear Cardiology
# RESULTS
### Chart
| Category | | | | |
|---|---|---|---|---|FIGURE 1. Comparison of stress MBF values on global and regional levels.
The X-axis is the difference in MBF values expressed in percent of corresponding medians; Y-axis is 1-ICC. The shaded iris area limits the acceptable agreement: ± 20% of the median value on the X-axis and ICCs over 0.75 on the Y-axis. The chart element corresponds to the comparison in the left ventricle area of the same name. C2 stands for Carimas, FQ for FlowQuant, PM for PMOD.
Copyright American Society of Nuclear Cardiology

## Slide 5
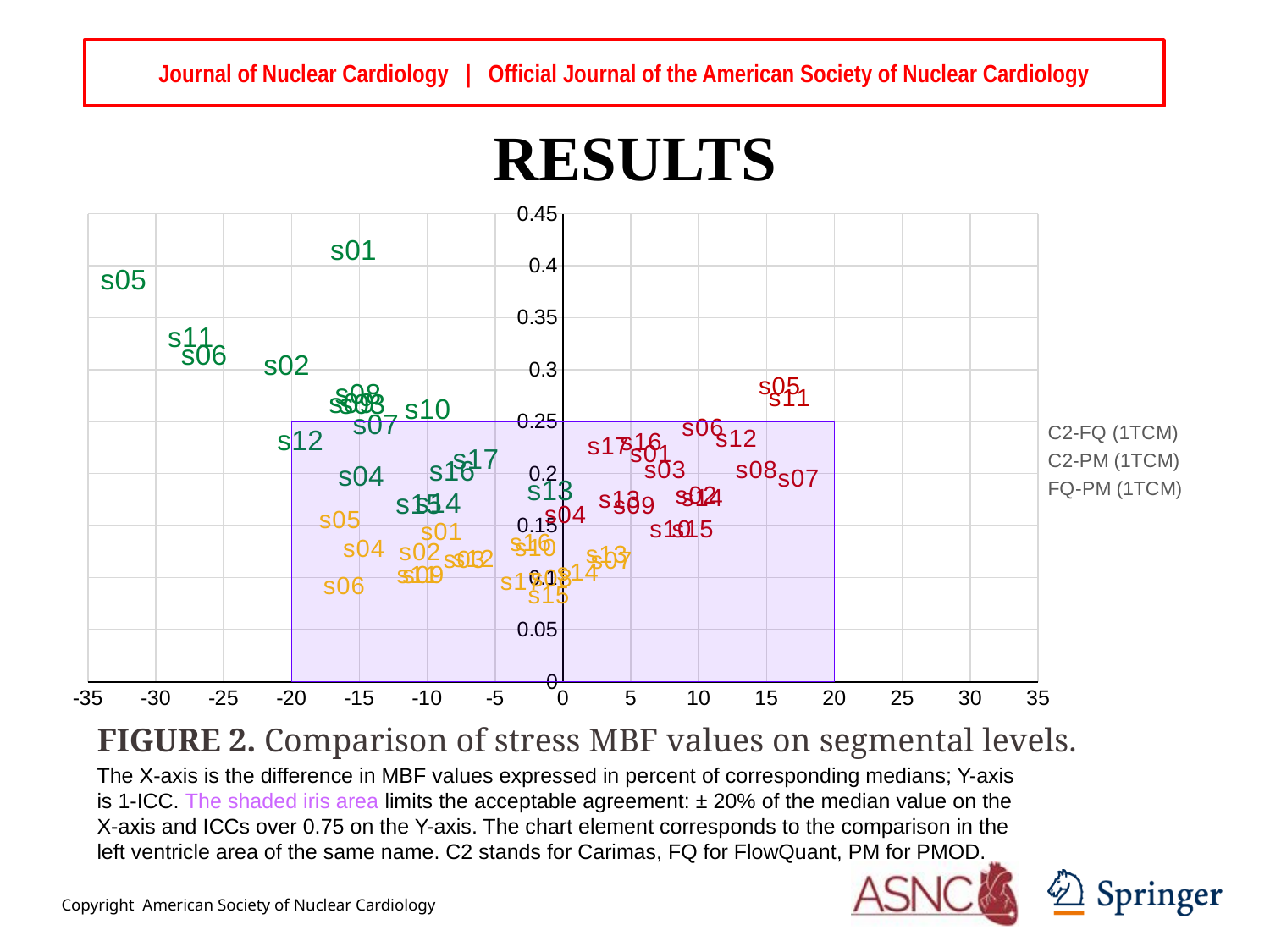

Journal of Nuclear Cardiology | Official Journal of the American Society of Nuclear Cardiology
# RESULTS
### Chart
| Category | | | | |
|---|---|---|---|---|FIGURE 2. Comparison of stress MBF values on segmental levels.
The X-axis is the difference in MBF values expressed in percent of corresponding medians; Y-axis is 1-ICC. The shaded iris area limits the acceptable agreement: ± 20% of the median value on the X-axis and ICCs over 0.75 on the Y-axis. The chart element corresponds to the comparison in the left ventricle area of the same name. C2 stands for Carimas, FQ for FlowQuant, PM for PMOD.
Copyright American Society of Nuclear Cardiology

## Slide 6
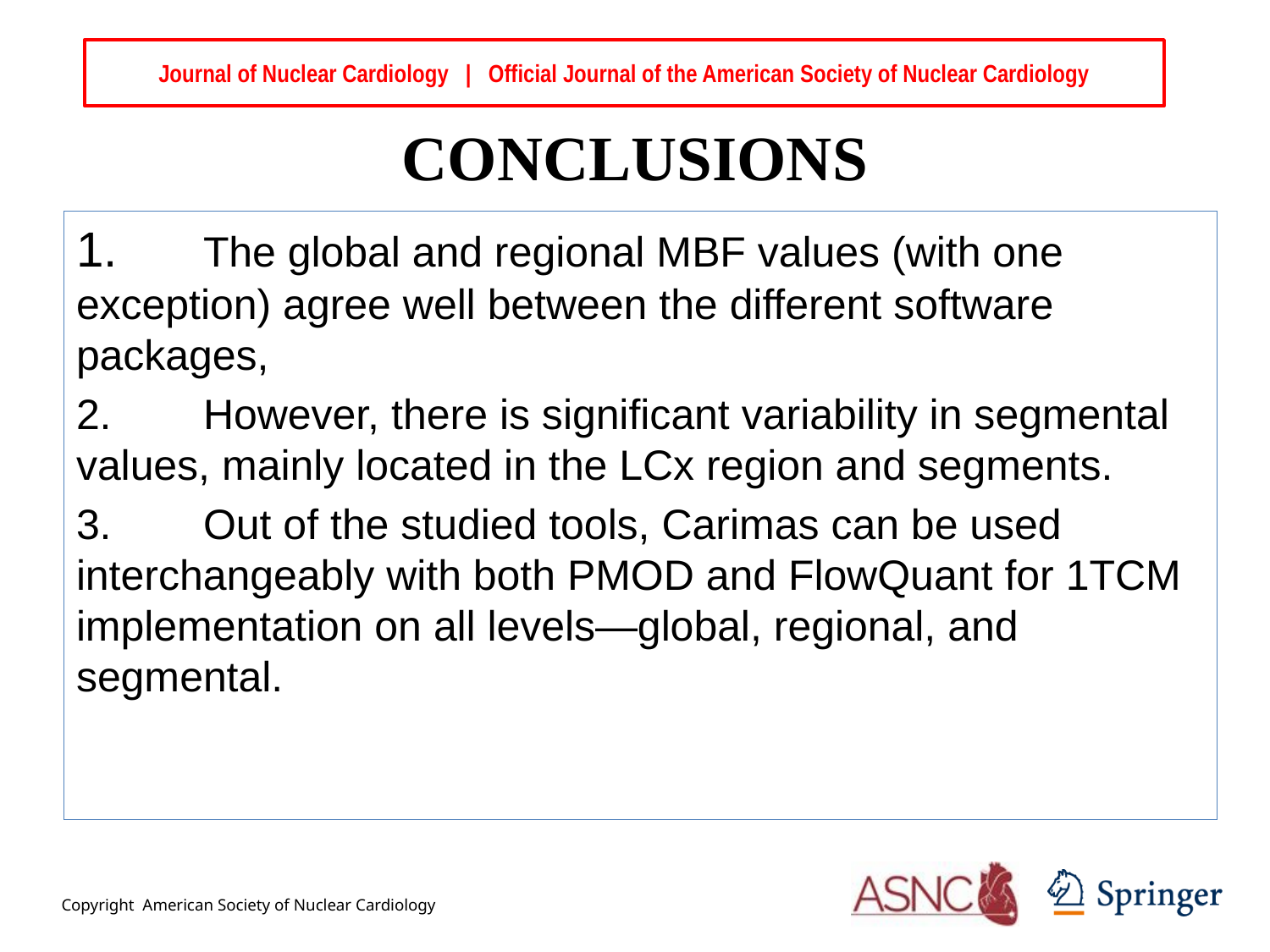

Journal of Nuclear Cardiology | Official Journal of the American Society of Nuclear Cardiology
# CONCLUSIONS
1.	The global and regional MBF values (with one exception) agree well between the different software packages,
2.	However, there is significant variability in segmental values, mainly located in the LCx region and segments.
3.	Out of the studied tools, Carimas can be used interchangeably with both PMOD and FlowQuant for 1TCM implementation on all levels—global, regional, and segmental.
Copyright American Society of Nuclear Cardiology
